# Deubiquitinylase USP47 Promotes RelA Phosphorylation and Survival in Gastric Cancer Cells

**DOI:** 10.3390/biomedicines6020062

**Published:** 2018-05-22

**Authors:** Lara Naghavi, Martin Schwalbe, Ahmed Ghanem, Michael Naumann

**Affiliations:** Institute of Experimental Internal Medicine, Otto von Guericke University, 39120 Magdeburg, Germany; lara.naghavi@med.ovgu.de (L.N.); martin.schwalbe@med.ovgu.de (M.S.); ahmed.ghanem@med.ovgu.de (A.G.)

**Keywords:** deubiquitinylases, NF-κB signaling, apoptotic cell death, gastric carcinoma, therapeutic targets, chemoresistance, ubiquitin-specific proteases

## Abstract

Every year, gastric cancer causes around 819,000 deaths worldwide. The incidence of gastric cancer in the western world is slowly declining, but the prognosis is unpromising. In Germany, the 5-year-survival rate is around 32%, and the average life span after diagnosis is 6 to 9 months. Therapy of gastric cancer patients comprises a gastrectomy and perioperative or adjuvant chemotherapy. However, resistance of gastric cancer cells to these agents is widespread; thus, improved chemotherapeutic approaches are required. Nuclear factor kappa B (NF-κB) transcription factors are associated with anti-apoptosis, carcinogenesis, and chemoresistance, and thus, constitute attractive targets for therapeutic intervention. In immunoblots, we show that ubiquitin specific protease 47 (USP47) promotes β-transducin repeat-containing protein (βTrCP) stability and phosphorylation of RelA. Furthermore, after knockdown of USP47 by RNA interference, we analyzed in gastric cancer cell lines metabolic activity/viability in an MTT assay, and apoptotic cell death by Annexin V staining and poly(ADP-Ribose) polymerase (PARP)-1, caspase 3, and caspase 8 cleavage, respectively. We found that USP47 contributes to cell viability and chemoresistance in NCI-N87 gastric carcinoma cells treated with etoposide and camptothecin. Inhibition of USP47 might be a suitable strategy to downregulate NF-κB activity, and to overcome chemoresistance in gastric cancer.

## 1. Introduction

With 1.3 million incident cases and 819,000 deaths recorded globally, gastric cancer was the fifth most common malignancy, and the third leading cause of cancer deaths in 2015 [1]. Surgical gastrectomy remains the only curative therapy. However, relapse occurs in 40–60% of cases [2]. Diagnosis of gastric cancers often occurs when tumors are inoperable and patients have median survival times of three to five months. First-line or adjuvant chemotherapy (or chemoradiation) extends patient survival times by 6–7 months [3]. The high number of deaths from gastric cancer, low cure rates, and tumor relapse after gastrectomy therefore demand the development of better chemotherapeutic agents to improve patient survival. In particular, overcoming chemoresistance in tumor cells that otherwise could result in relapse or metastasis poses a major challenge [4,5].

Nuclear factor kappa B (NF-κB) constitutes a family of transcription factors (RelA, RelB, c-Rel, p105/p50 and p100/52) that regulate many target genes involved in inflammation, immunity, cell proliferation, or cell survival. Without stimulation, NF-κB molecules are sequestered to the cytoplasm by inhibitors of kappa B (IκB) molecules. Stimulation of the canonical NF-κB pathway results in the activation of the IκB kinase (IKK) complex, which in turn phosphorylates IκBα, promoting its ubiquitinylation and subsequent degradation. After release from IκBα, NF-κB factors translocate to the nucleus stimulating transcription of their target genes. Transcriptional activity of NF-κB is further regulated by posttranslational modifications like phosphorylation, ubiquitinylation, or acetylation [6]. Importantly, dysregulation of NF-κB signaling promotes carcinogenesis [5,7,8] and chemoresistance [9,10,11].

As novel therapeutic targets, deubiquitinylases are receiving increased attention, due to their association with cancer and neurodegenerative diseases [12,13]. Deubiquitinylases (DUBs) as antagonists to E3 ubiquitin ligases are an integral part of the ubiquitin-proteasome-system. E3s conjugate ubiquitin moieties to substrate proteins, and thus, regulate their stability, protein interactions, or subcellular localization. In contrast, to the more than 600 E3 ligases [14] in humans with distinct substrate specificities, less than 100 DUBs reverse the conjugation and cleave ubiquitin moieties from substrate proteins. In particular, within the NF-κB pathway, E3s and DUBs regulate IKK complex activation, IκBα degradation, or NF-κB activity [15,16,17]. Because the NF-κB pathway constitutes an attractive target for therapeutic intervention in gastric cancer [5,18], we performed an siRNA screen aiming to identify potential DUB targets that are essential for NF-κB activity. As one such target, we identified the ubiquitin specific protease 47 (USP47), that was previously shown to be essential for the proliferation of gastric carcinomas [19]. Furthermore, depletion of USP47 sensitized osteosarcoma and breast cancer cell lines towards drug and UV radiation-induced apoptosis [20].

In this study, we addressed the molecular details of NF-κB regulation using USP47, and examined the viability of USP47 as a promising target for drug intervention to enhance the action of current drugs, and to overcome chemoresistance in gastric cancer cells. Our results show that depletion of USP47 in AGS gastric cancer cells results in decreased protein levels of phospho-RelA and β-transducin repeat-containing protein (βTrCP). Even though USP47 depletion failed to increase apoptotic cell death in AGS cells treated with camptothecin (CPT) and etoposide (Eto), it overcame chemoresistance in NCI-N87 gastric carcinoma cells. Therefore, targeting of USP47 represents a suitable strategy to overcome drug resistance in gastric carcinomas.

## 2. Experimental Section

### 2.1. Cell Culture and siRNA Transfection

Gastric carcinoma cell lines AGS and NCI-N87 (ATCC, LGC Standards GmbH, Wesel, Germany) were cultured in an RPMI 1640 medium, supplemented with 10% fetal calf serum (FCS) and 100 U/mL penicillin/streptomycin, and then incubated at 37 °C in a 5% CO_2_ humidified incubator. AGS and NCI-N87 cells were seeded at a density of 1.2 or 1.5 × 10^6^, respectively, per 100 mm culture dish (CellStar, Greiner Bio-one GmbH, Frickenhausen, Germany). For transient knockdown of USP47, cells at 30% confluency were transferred into serum-free OptiMEM medium (Thermo Fisher Scientific, Waltham, MA, USA), and transfected with 50 nM siRNA that had been mixed with 10 μL/10 cm culture dish SiLentFect Lipid Reagent (Bio-Rad, Hercules, CA, USA). USP47 siRNA 5′-CUAUAACUGUUCGUGCUUA-3′ was obtained from Eurofins Genomics, Ebersberg, Germany, and AllStars negative control siRNA from Qiagen, Hilden, Germany. Six hours after transfection, a fresh RPMI 1640 medium with 10% FCS was added, and incubation continued for 42 h. Sixteen hours prior to TNF treatment, cells were serum starved with an RPMI 1640 medium supplemented with 0.2% FCS; 10 ng/mL TNF (in PBS) (PeproTech, Hamburg, Germany) was used, as described previously [17]. Camptothecin or etoposide (all Sigma-Aldrich, St. Louis, MO, USA) were used at concentrations of 1 μM (in DMSO) or 10 μM (in DMSO), respectively.

### 2.2. Subcellular Fractionation

Cells were washed twice with PBS and scraped into chilled 1 mL PBS. After removing the PBS by centrifugation (2 min, 1000× *g*), the cell pellet was suspended in 400 μL chilled buffer A (20 mM Tris pH 7.9, 10 mM NaCl, 1.5 mM MgCl_2_, 10% glycerol, 1 mM DTT, 0.5 mM AEBSF, 1 mM Na_3_VO_4_, 1 mM Na_2_MO_4_, 10 mM NaF, 10 mM K_2_HPO_4_, 20 mM 2-Phosphoglycerate, 7.5 mM N-Ethylmaleimid (NEM), 5 mM ortho-phenanthroline (OPT), 1× cOmplete EDTA-free protease inhibitor cocktail (Roche Diagnostics, Mannheim, Germany)), and incubated for 10 min on ice. Subsequently, 4 μL NP40 was added to lyse the cells. After 5 min incubation on ice, nuclei were separated from cytosolic supernatants by centrifugation (10 min, 2000× *g*). Cytosolic supernatants were clarified by centrifugation (10 min, 13,000× *g*), and nuclear pellets washed with 100 μL buffer A. Nuclear pellets were suspended in 50 μL buffer C (20 mM Tris pH 7.9, 420 mM NaCl, 1.5 mM MgCl_2_, 10% glycerol, 0.2 mM EDTA, 0.5 mM DTT, 0.5 mM AEBSF, 1 mM Na_3_VO_4_, 1 mM Na_2_MO_4_, 10 mM NaF, 10 mM K_2_HPO_4_, 20 mM 2-Phosphoglycerate, 7.5 mM NEM, 5 mM OPT, 1× cOmplete EDTA-free protease inhibitor cocktail), and incubated for 30 min on ice, with occasional vortexing. Soluble nuclear proteins (N1) were obtained after centrifugation (10 min, 13,000× *g*). The BCA assay (Thermo Fisher Scientific) was used to determine protein concentration.

### 2.3. SDS-PAGE and Immunoblotting

Proteins were separated by SDS-PAGE in Tris-Glycine buffer gels and transferred onto PVDF membranes (Merck, Darmstadt, Germany), followed by 1 h blocking at room temperature using 5% skim milk in TBS containing 0.1% Tween (TBS-T). The PVDF membranes were incubated overnight at 4 °C, with the primary antibodies in either 5% BSA or 5% skim milk/TBS-T. The membranes were washed thrice with TBS-T, and incubated with the appropriate HRP-conjugated secondary antibody diluted 1:5000 in 5% skim milk/TBS-T for 1 h at room temperature. All antibodies used in this study are listed in the Supplementary Materials Table S1. After washing the membranes thrice with TBS-T, the blots were developed using a chemiluminescent substrate (Merck), and protein bands were visualized using the ChemoCam Imager (Intas, Göttingen, Germany).

### 2.4. Apoptosis Detection

Forty-eight hours after transfection, cells were treated for 24 h with either camptothecin or etoposide. Control cells were treated with equal volumes of DMSO. Externalization of phosphatidylserine was determined 24 h after treatment using the Annexin V-FITC kit (Biotool, Houston, TX, USA). Cells were labeled with Annexin V-FITC according to instructions, and apoptotic cells (Annexin V-FITC positive) were counted by flow cytometry (CyFlow space, Partec, Görlitz, Germany). Whole cell extracts for immunoblotting were prepared by suspending the cells in 100 μL RIPA buffer (50 mM Tris, pH 7.5, 150 mM NaCl, 10 mM K_2_HPO_4_, 5 mM EDTA, 10% glycerol, 1% Triton X100, 0.05% SDS, 1 mM AEBSF, 20 mM 2-phosphoglycerate, 20 mM NaF, 1 mM Na_3_VO_4_, 1 mM Na_2_MoO_4_, 1× cOmplete EDTA-free protease inhibitor cocktail), followed by 10 min incubation on ice, and subsequent clarification of the extract (10 min, 13,000× *g*).

### 2.5. Cell Viability Assay

Cells were seeded in 96-well plates with 50,000 cells per well, and transfected as described above. Twenty-four hours after camptothecin or etoposide, treatment viability was assessed using the MTT Cell Viability Assay Kit (Abnova, Taipei, Taiwan). Assays were performed according to instructions, and absorption was measured at 570 nm on a Spectramax M5 plate reader (Molecular Devices GmbH, Biberach an der Riss, Germany). The average absorption value for the control cells was set to 100% viability.

### 2.6. Statistics

All quantitative data were presented as mean ± S.D (standard deviation). Statistical analysis was performed using Student’s *t*-test (Excel, Microsoft Office Plus 2010). *p* < 0.05 was regarded as significant.

## 3. Results

### 3.1. Depletion of USP47 Decreased Phospho-RelA and βTrCP Protein Levels

**Figure 1:**
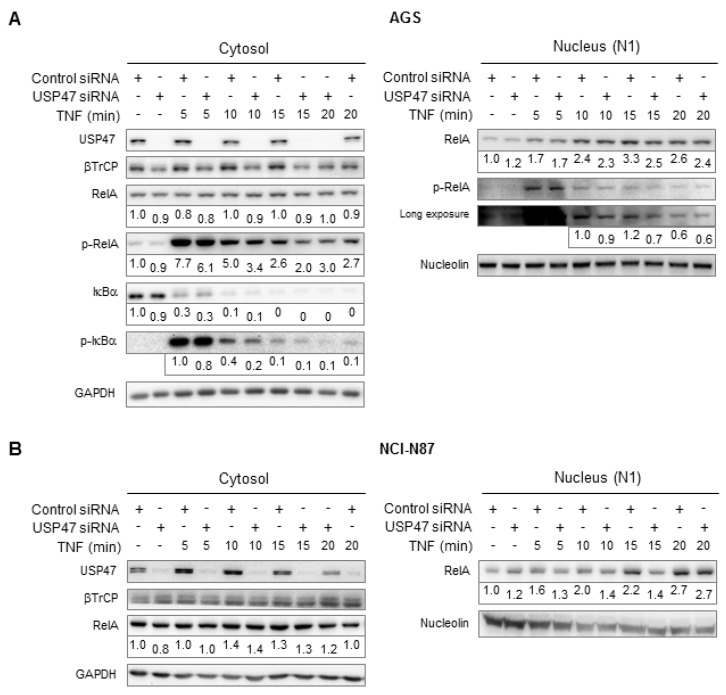
USP47 depletion downregulates protein levels of βTrCP and Ser536-phosphorylated RelA. (**A**) AGS or (**B**) NCI-N87 cells transfected with USP47 or a negative control siRNA were stimulated with TNF for up to 20 min. Subcellular fractions were prepared at the indicated time points and NF-κB pathway components analyzed in cytosolic and soluble nuclear fractions (N1). An additional immunoblot with increased signal acquisition time is shown for p-RelA in (**A**). Detection of GAPDH and nucleolin served as control for equal protein load. For densitometric analysis, band intensities of key proteins were normalized to the band intensities of the respective cytosolic or nuclear loading control. The normalized band intensities are shown below the corresponding blots.

**Figure 2:**
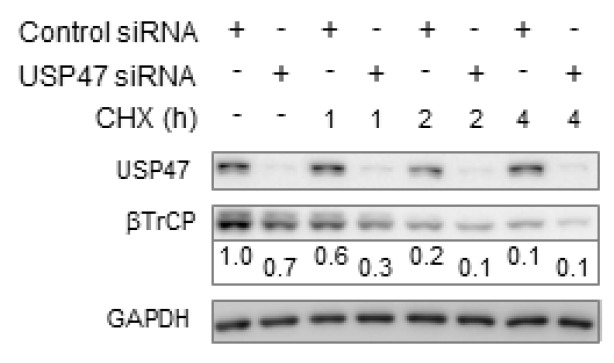
USP47 depletion downregulates basal βTrCP protein levels. AGS cells transfected with USP47 or a negative control siRNA were treated with CHX for up to 4 h. Subcellular fractions were prepared at the indicated time points and βTrCP stability was analyzed in the cytosolic fractions.

Based on the observation that USP47 promotes the proliferation of gastric carcinomas [19], we performed a USP47 knockdown in AGS gastric carcinoma cells. Stimulation with tumor necrosis factor (TNF) for up to 20 min in USP47 knockdown cells resulted in decreased Ser536-phosphorylated RelA protein levels in the cytosol, and to a minor extent, also in the nucleus (Figure 1A). In contrast, IκBα degradation appeared unaffected, even though a minor decrease in IκBα phosphorylation was noticed after USP47 knockdown (Figure 1A). Analysis of regulatory NF-κB pathway components revealed that USP47 depletion constitutively decreased βTrCP levels (Figure 1A). βTrCP is the substrate adaptor of the E3 ligase complex that facilitates IκBα ubiquitinylation after phosphorylation. Inhibition of protein translation through cycloheximide (CHX) treatment showed that USP47 depletion decreased the basal expression levels of βTrCP (Figure 2). Similar to AGS cells, knockdown of USP47 in gastric cancer cell line (NCI-N87) constitutively decreased βTrCP levels, and reduced nuclear translocation of RelA (Figure 1B).

### 3.2. USP47 Promotes Chemoresistance and Cell Viability in NCI-N87 Gastric Carcinoma Cells

**Figure 3:**
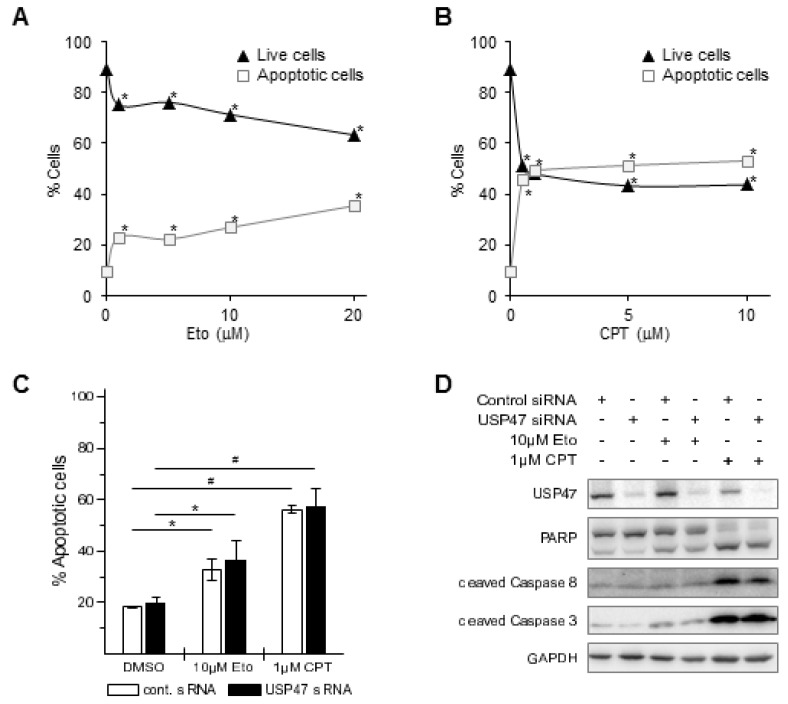
Etoposide (Eto) or camptothecin (CPT) induce apoptosis in AGS cells. 24 h after incubation of AGS cells with increasing amounts of (**A**) Eto (1–20 μM) or (**B**) CPT (0.5–10 μM) live (black triangles) and apoptotic cells (open squares) were counted by flow cytometry using Annexin V staining. Standard deviations are in the range of the marker size. * *p* < 0.05. USP47 depletion does not enhance Eto or CPT-induced apoptosis in AGS cells. AGS cells were incubated with 10 μM Eto or 1 μM CPT for 24 h alone or in combination with USP47 knockdown; (**C**) Apoptotic cells were counted by flow cytometry using Annexin V staining. * *p* < 0.05, # *p* < 0.001; (**D**) Whole cell extracts were subjected to immunoblotting and analyzed for apoptotic markers. Detection of GAPDH served as control for equal protein load.

**Figure 4:**
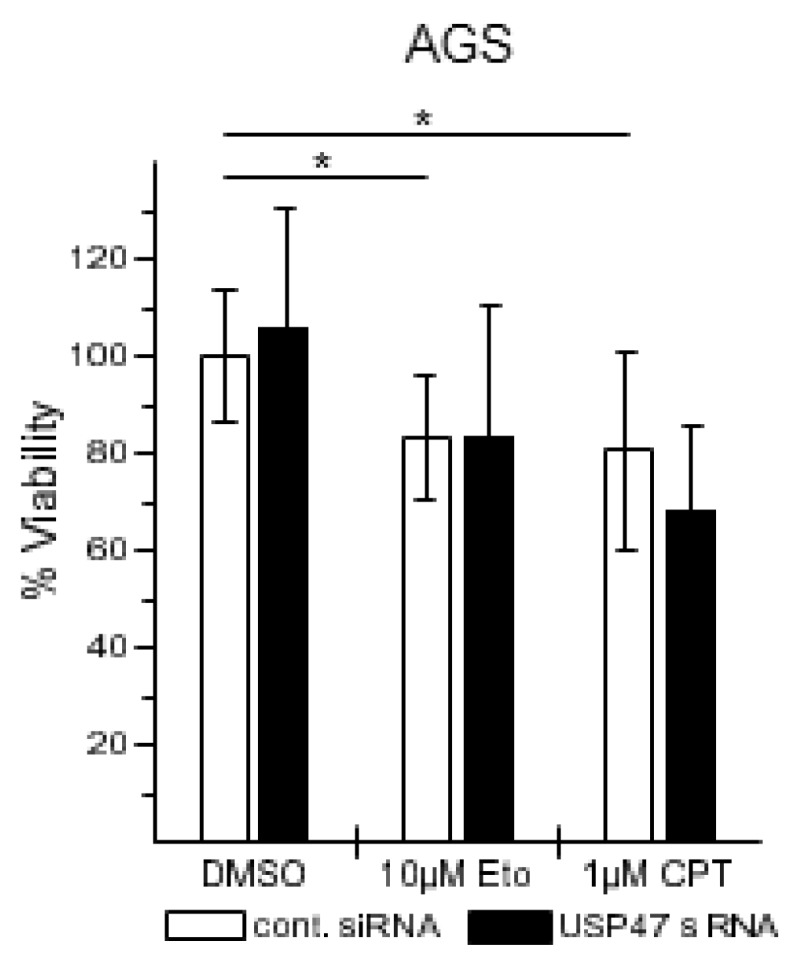
USP47 promotes cell viability in AGS cells. AGS cells were seeded in 96 well plates, transfected with control or USP47 siRNA and incubated for 24 h with either with 10 μM Eto or 1 μM CPT. Cell viability was measured using the 3-(4,5-Dimethylthiazol-2-yl)-2,5-diphenyltetrazoliumbromid (MTT) assay. * *p* < 0.05.

TNF can induce diverse cellular responses, including cell survival and apoptosis [21]. Gastric cancer patients have low cure rates after chemotherapy; we therefore asked whether USP47 knockdown could enhance apoptosis in gastric cancer cells when treated with chemotherapeutic drugs. We selected the topoisomerase I and II inhibitors CPT and Eto to treat different gastric carcinoma cell lines, because their mode of action is similar to drugs used in current chemotherapy regimens [3], and because they are known to also promote NF-κB-dependent apoptosis resistance [22,23]. Eto or CPT treatment of AGS cells increased apoptotic cell death by ca. 18% or 38% in a concentration dependent-manner, respectively, (Figure 3A–C), promoted caspase 8, caspase 3, and poly(ADP-Ribose) polymerase (PARP)-1 cleavage (Figure 3D), and decreased cell viability by ca. 20% (Figure 4). Combining USP47 knockdown with Eto or CPT treatment did not further enhance apoptotic cell death, and resulted in comparable levels of apoptotic cells and cell viability (Figure 3C,D and Figure 4).

**Figure 5:**
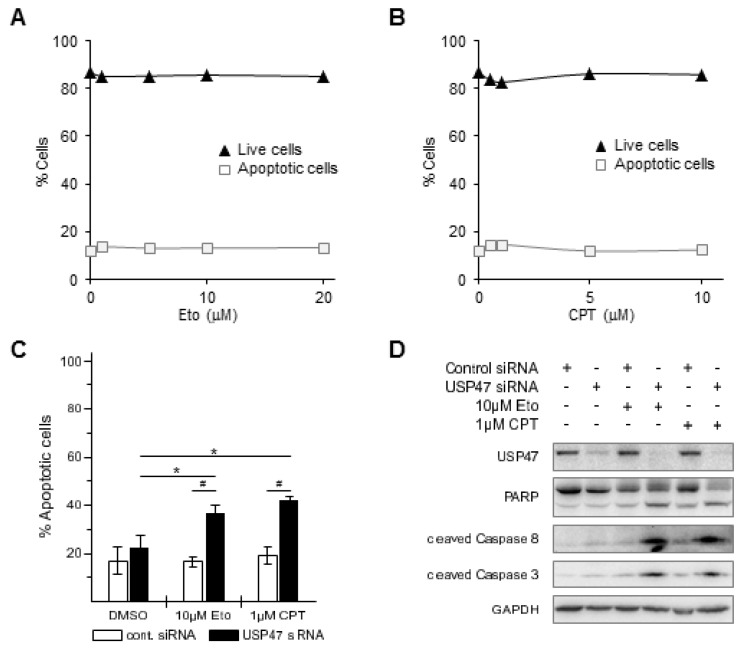
USP47 attenuates etoposide (Eto) or camptothecin (CPT)-induced apoptotic cell death in NCI-N87 cells. NCI-N87 cells were incubated with increasing concentrations of (**A**) Eto (1–20 μM) or (**B**) CPT (0.5–10 μM) for 24 h. Live (black triangles) and apoptotic cells (open squares) were counted by flow cytometry using Annexin V staining. Standard deviations are in the range of the marker size; (**C**) To analyze the effect of USP47 on cell survival, NCI-N87 cells were transfected with USP47 or control siRNA, treated for 24 h with 10 μM etoposide or 1 μM CPT and apoptotic cells were counted by flow cytometry using Annexin V staining. * *p* < 0.05, # *p* < 0.001; (**D**) Whole cell extracts of NCI-N87 cells treated as in (**C**) were subjected to immunoblotting and analyzed for apoptotic markers. Detection of GAPDH served as control for equal protein load.

**Figure 6:**
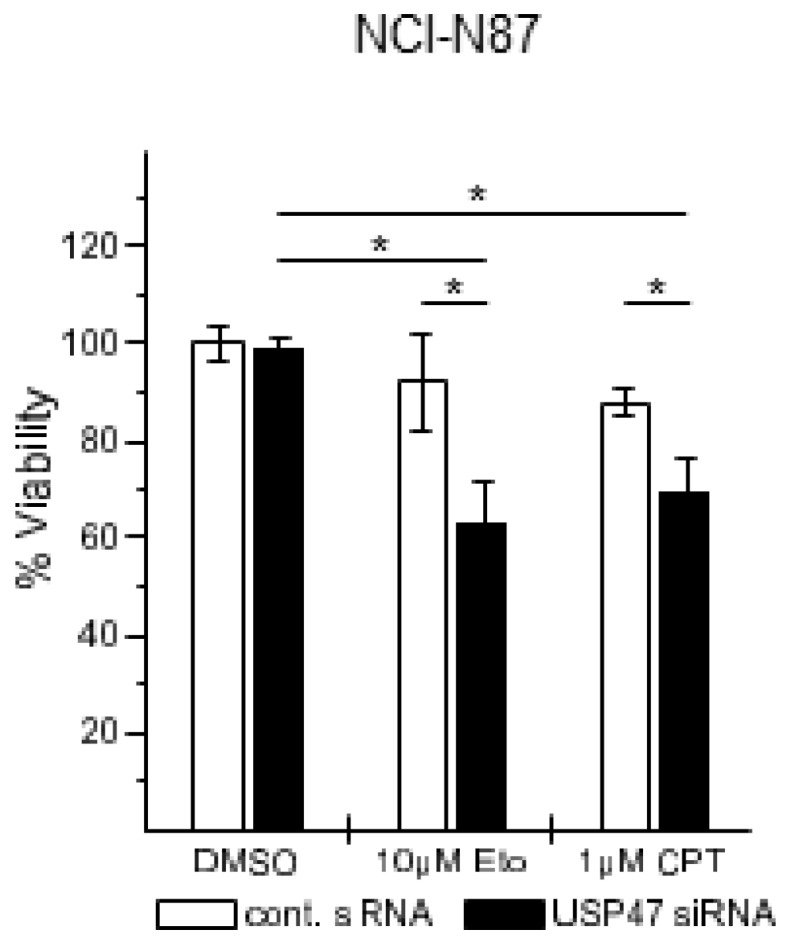
USP47 promotes cell viability in NCI-N87 cells. NCI-N87 cells were seeded in 96 well plates, transfected with control or USP47 siRNA and incubated for 24 h with either with 10 μM Eto or 1 μM CPT. Cell viability was measured using the 3-(4,5-Dimethylthiazol-2-yl)-2,5-diphenyltetrazoliumbromid (MTT) assay. * *p* < 0.05.

Analysis of additional gastric carcinoma cell lines showed that the NCI-N87 cell line was resistant to Eto and CPT treatment, independent of the concentration (Figure 5A,B). Interestingly, after USP47 knockdown, the NCI-N87 cell line displayed increased apoptotic cell death (Figure 5C), increased cleavage of PARP, caspase 3, and caspase 8 (Figure 5D), and reduced cell viability (Figure 6).

## 4. Discussion

After cardiovascular diseases, cancer is the second leading cause of death worldwide, and with an increasing and ageing population, it is expected that patient numbers will rise [1]. Chemoresistance constitutes a major problem of chemotherapy, because it promotes relapse and thus increases morbidity [4]. Therefore, it is necessary to improve current chemotherapy regimens and identify new drug targets, especially for malignancies like gastric carcinomas with low cure rates [3]. Deubiquitinylases represent promising novel targets for drug discovery in cancer therapy, due to their involvement in metastasis and their regulatory role in apoptosis pathways [24]. In this study we could identify USP47 as a regulator of the NF-κB pathway. Therapeutic intervention of NF-κB signaling is an intensively studied research field [18,25], because aberrant NF-κB activation in the context of cancer promotes carcinogenesis, tumor progression, metastasis, or chemoresistance [5].

Our results demonstrate that USP47 stabilizes the E3 ligase complex substrate adaptor protein βTrCP, and promotes phosphorylation of RelA at Ser536 (Figure 1). The residual βTrCP levels after USP47 depletion suffice to allow for IκBα degradation. Within the chosen time points, differences between USP47 and control cells did not become apparent, probably due to the rapidness of the process. Nonetheless, nuclear translocation of RelA is diminished after USP47 knockdown (Figure 1), which is consistent with a delayed release from IκBα.

Apart from its regulatory role in the NF-κB pathway, USP47 constitutes an interesting target for gastric cancer chemotherapy, because it promotes gastric carcinoma cell proliferation [19], and its depletion sensitize cancer cells to chemotherapy, probably due to the upregulation of Cdc25a [20]. We therefore addressed the question of whether USP47 inhibition could affect cell survival/proliferation of gastric cancer cells. For instance, USP47 inhibition could be beneficial in attenuating NF-κB-dependent chemoresistance in response to DNA double strand break inducing agents [22]. AGS cells displayed pronounced apoptotic cell death when treated with CPT or Eto (Figure 3A–D), but no further enhancement was observed after USP47 depletion. It is likely that the already strong response masks additional contributions to the apoptosis caused by the USP47 knockdown. Interestingly, in the CPT- and Eto-resistant gastric carcinoma cell line NCI-N87 (Figure 5A,B) USP47 depletion resulted in pronounced apoptosis induction after CPT and Eto treatment (Figure 5C,D and Figure 6). The molecular mechanisms responsible for the drug-resistance are currently unknown. However, it is likely that in addition to the attenuation of NF-κB activity, other factors involved in DNA double strand break repair might play a role. For instance, USP47 stabilizes DNA polymerase β, and hence regulates base excision repair after DNA damage [26]. Similarly, USP47 might stabilize protein factors involved in DNA double strand break repair, and hence, depletion of USP47 could sensitize cancer cells to the action of DNA damaging agents.

USP47 depletion on its own caused no increase in apoptotic cell death (Figure 3, Figure 4, Figure 5 and Figure 6), suggesting that loss of USP47 activity is not cytotoxic. This together with its potential to overcome chemoresistance in gastric carcinoma cells suggests that USP47 could represent a promising therapeutic target structure. Furthermore, a combination of current chemotherapy regimens together with USP47 inhibition might allow the reduction of the concentration of chemotherapeutic agents without impairing the cytotoxic effect on cancer cells, while reducing side effects. We propose that the development of USP47 inhibitors and their combination with current chemotherapy regimens might reduce relapse after gastrectomy, and extend overall patient survival times.

## 5. Conclusions

Here, we could show that USP47 regulates NF-κB activity by promoting the phosphorylation of RelA through the stabilization of βTrCP and subsequent degradation of IκBα. In addition, USP47 contributes to chemoresistance and viability in NCI-N87 gastric carcinoma cells. Inhibition of USP47 activity could represent a viable strategy for gastric cancer chemotherapy by downregulating transcription of NF-κB-regulated pro-survival genes, and overcoming NF-κB-dependent chemoresistance.

## Supplementary Materials

The following are available online at http://www.mdpi.com/2227-9059/6/2/62/s1, Table S1: List of primary and secondary antibodies: Uncropped immunoblots corresponding to data shown in this study.
