# Supplementary material for: Deubiquitinylase USP47 Promotes RelA Phosphorylation and Survival in Gastric Cancer Cells
Source: Biomedicines. 2018 May 22;6(2):62. doi: 10.3390/biomedicines6020062 (PMC6027160; doi:10.3390/biomedicines6020062)
Supplement: Supplementary file 1 [file biomedicines-06-00062-s001.pdf]

Supplementary Information

Deubiquitinylase USP47 promotes RelA phosphorylation and survival in gastric cancer cells

Lara Naghavi[1$] , Martin Schwalbe[1$], Ahmed Ghanem[1] and Michael Naumann[1*]

[1] Institute of Experimental Internal Medicine, Otto von Guericke University, 39120 Magdeburg, Germany; lara.naghavi@med.ovgu.de (L.N.); martin.schwalbe@med.ovgu.de

***** Correspondence: Naumann@med.ovgu.de; Tel.: +49-391-67-13227

Table S1. List of primary and secondary antibodies used in the study

Antibody	Host species	Supplier	Cat. no.
Actin	goat	Santa Cruz Biotechnology	sc1616
βTrCP	rabbit	Cell Signaling Technology	#4394
Caspase 3, cleaved	rabbit	Cell Signaling Technology	#9661
Caspase 8	mouse	Cell Signaling Technology	#9746
GAPDH	mouse	Millipore	MAB374
IκBα	rabbit	Santa Cruz Biotechnology	sc371
IκBα, phospho S32/S36	mouse	Cell Signaling Technology	#9246
PARP-1	rabbit	Cell Signaling Technology	#9542
RelA	rabbit	Santa Cruz Biotechnology	sc372
RelA, phospho S536	rabbit	Cell Signaling Technology	#3031
Ubiquitin	mouse	Cell Signaling Technology	sc8017
USP47	rabbit	abcam	ab72143
Goat IgG	rabbit	Sigma-Aldrich	#A5420
Mouse IgG	goat	Jackson Immuno research	#115-035-174
Rabbit IgG	mouse	Jackson Immuno research	#211-032-171

Uncropped immunoblots corresponding to data shown in Figures 1, 2, 3D and 5D.

Figure 1A

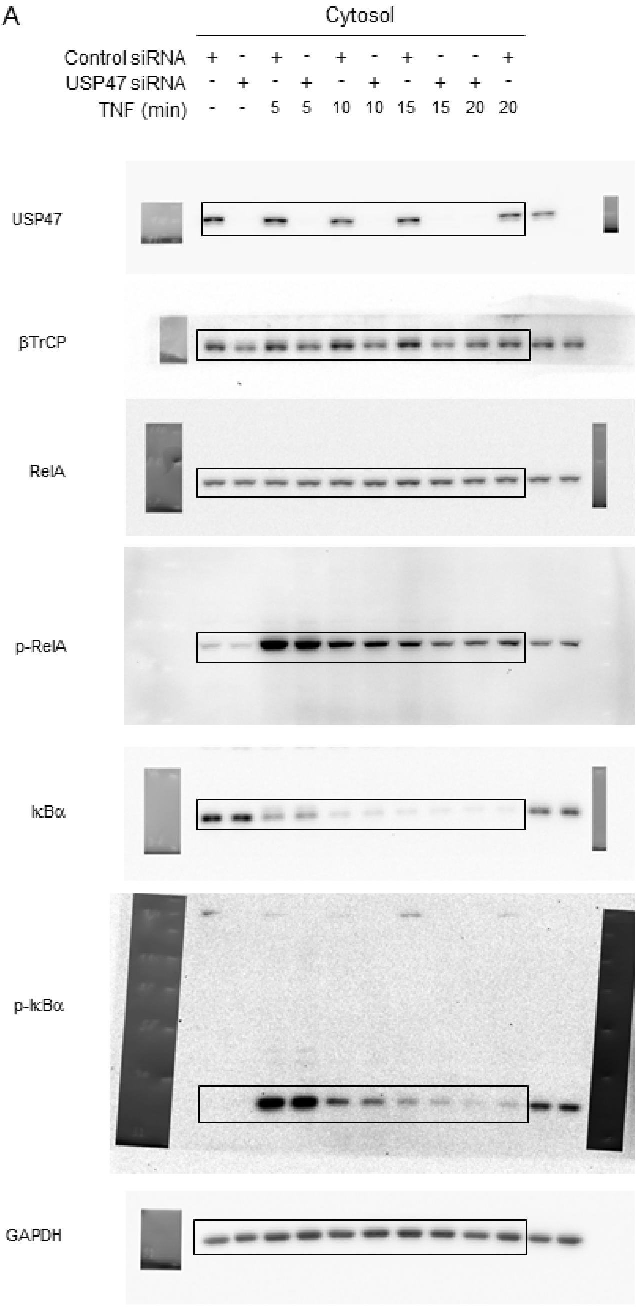
Cytosol
Control siRNA + - + - + - + - - +
USP47 siRNA - + - + - + - + + -
TNF (min) - - 5 5 10 10 15 15 20 20
USP47
βTrCP
RelA
p-RelA
IκBα
p-IκBα
GAPDH

Figure 1A

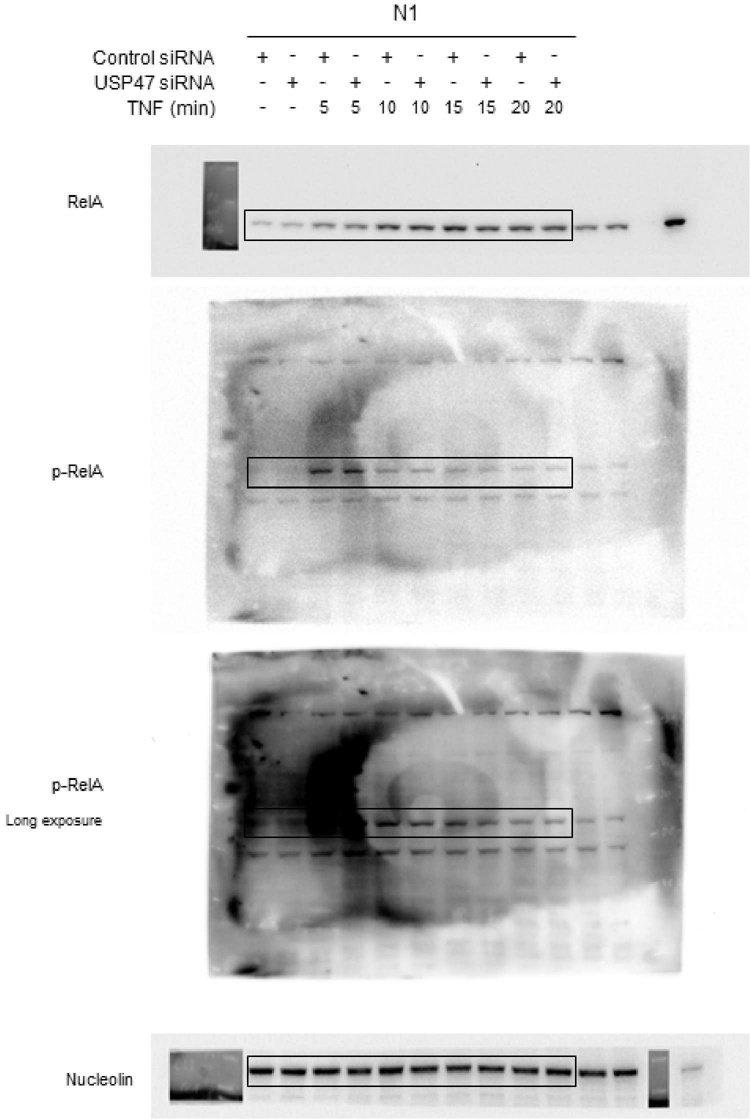
N1
Control siRNA + - + - + - + - + -
USP47 siRNA - + - + - + - + - +
TNF (min) - - 5 5 10 10 15 15 20 20
RelA
p-RelA
p-RelA
Long exposure
Nucleolin

Figure 1B

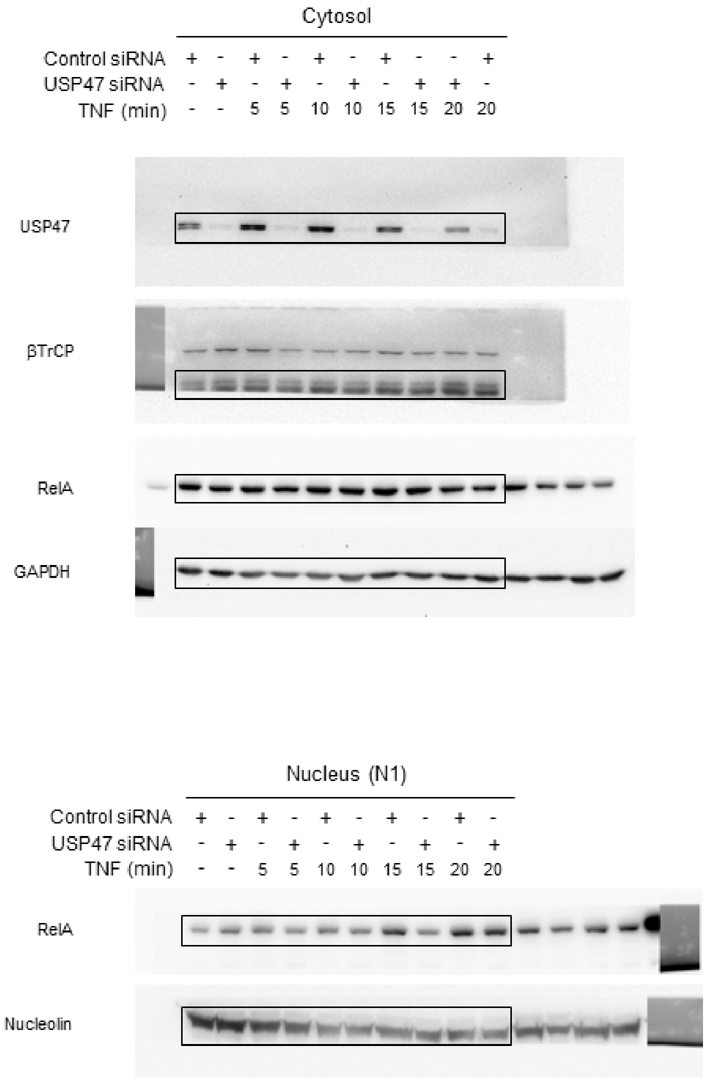
Cytosol
Control siRNA + - + - + - + - - +
USP47 siRNA - + - + - + - + + -
TNF (min) - - 5 5 10 10 15 15 20 20
USP47
βTrCP
RelA
GAPDH
Nucleus (N1)
Control siRNA + - + - + - + - + -
USP47 siRNA - + - + - + - + - +
TNF (min) - - 5 5 10 10 15 15 20 20
RelA
Nucleolin

Figure 2

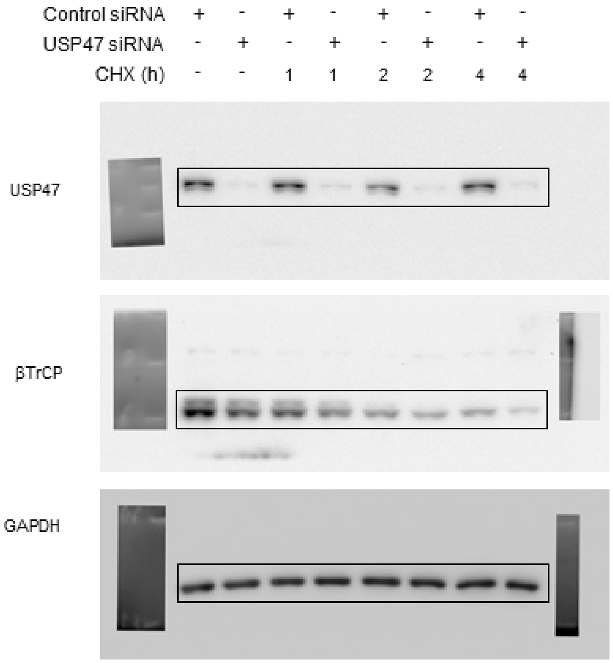

Figure 3D

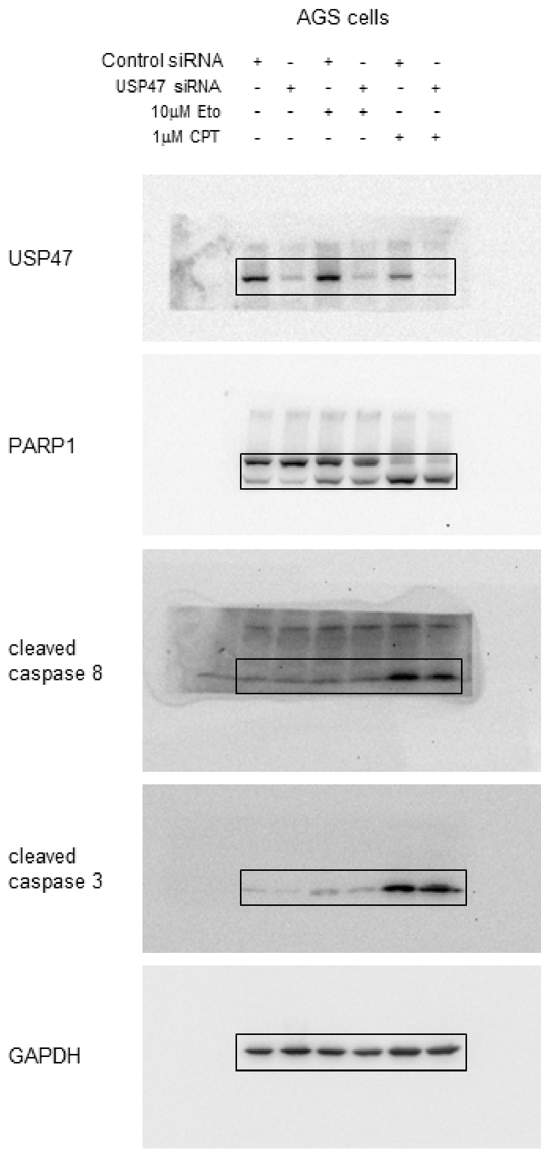
AGS cells
Control siRNA + - + - + -
USP47 siRNA - + - + - +
10μM Eto - - + + - -
1μM CPT - - - - + +
USP47
PARP1
cleaved caspase 8
cleaved caspase 3
GAPDH

Figure 5D

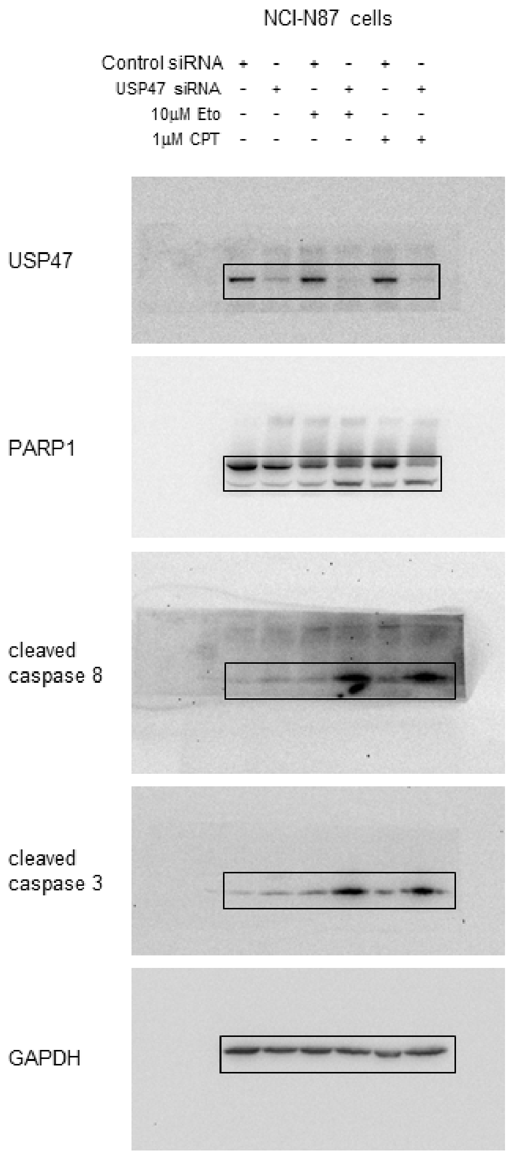
NCI-N87 cells
Control siRNA + - + - + -
USP47 siRNA - + - + - +
10μM Eto - - + + - -
1μM CPT - - - - + +
USP47
PARP1
cleaved caspase 8
cleaved caspase 3
GAPDH
